# Alcohol abusive use increases facial trauma?

**DOI:** 10.4317/medoral.21011

**Published:** 2016-07-31

**Authors:** Suzana-Célia-de-Aguiar Soares-Carneiro, Belmiro-Cavalcanti Vasconcelos, Gessyca-Suielly-Melo Matos da-Silva, Luciano-Cruz de-Barros-Caldas, Gabriela-Granja Porto, Jefferson-Figueiredo Leal, Ivson Catunda

**Affiliations:** 1 Postgraduate Student of the Doctor. Degree Program, Department of Oral and Maxillofacial Surgery, University of Pernambuco, Recife, PE, Brazil; 2 Adjunct Professor. Department of Oral and Maxillofacial Surgery, University of Pernambuco, Recife-PE; 3 Graduate Student of Dentistry. University of Pernambuco, Recife-PE; 4 Adjunct Professor. Department of Forensic Sciense, University of Pernambuco, Recife-PE

## Abstract

**Background:**

Trauma is among the main death causes and morbidity in the world and is often related to the use of alcohol and its abuse has reached massive proportions, no matter if the country is developed or not, being considered as public health problem. Since there are very few randomized and prospective studies in literature about the association of facial trauma and the use of alcohol, this study aims to investigate the impact of alcohol use in facial trauma.

**Material and Methods:**

This was a prospective and cross sectional study, involving facial trauma patients attended at Oral Maxillofacial Surgery Division of a State Hospital. Variables included patient´s profile, trauma etiology, facial region involved, type of injury and treatment and days of hospitalization. AUDIT test was applied to identify risks and damages of alcohol use and chemical dependence. Absolute distribution, uni and mutilvaried percentages were made for data evaluation. Pearson´s qui-squared and Fisher´s Exact tests were also used.

**Results:**

One hundred patients were evaluated. The patient´s mean age was 33.50 years-old, 48% had between 17 and 29 years old, 28% had 30 to 39, and 24% 40 or more. Most of them were male (86%). The most frequent etiology was traffic accident (57%), the extraoral area was most committed (62%), the most frequent type of injury was fractures (78%) and the most affected bone was the mandible (36%). More than half of the patients (53%) had surgical treatment. 38% had their discharge from hospital right after the first attendance. The AUDIT most frequent answer was “moderate use” (46%) and use at risk (39%). There was significant difference between the use of alcohol (AUDIT) and hematoma (0.003) and number of days of hospitalization (*p*=0.005).

**Conclusions:**

In this study it was not observed association between alcohol consumption using the AUDIT and trauma etiology, but patient victims of traffic accidents were classified as with risk in the scale. Most of the trauma were caused by traffic accidents using motorcycles and occurred in young aged men.

**Key words:**Wounds and injuries, traumatology and alcohol-induced disorders.

## Introduction

According to WHO, road injury is among the main death causes and morbidity in the world; in 2012, around 1,3 million people die of some kind of trauma caused by road injury. Brazil is the fifth in the world in number of traffic´s death. In 2010, there were 43,869 deaths according to the World Health Organization. This number, in absolute values, is inferior only to three other countries: India (231,077); China (275,983); and Nigeria (53,339). Therefore Brazil is at an intermediary position in a ranking between countries with 22.5 deaths for each 100,000 habitants (1).

Trauma is often related to the use of alcohol and its abuse has reached massive proportions (2), no matter if the country is developed or not, being considered as public health problem. Furthermore alcohol has a strong association with facial injuries due to interpersonal violence and motor vehicle accidents (1,3). It has also been showed alcohol interferes in cognitive and motor answers, prejudices capability for solving problems in conflict situation. Due to these effects, there is a direct correlation between alcohol consumption and the risk of a person being involved in a dangerous situation that may cause facial trauma, such as car accidents and interpersonal violence (1). However, relatively little is known, however, about the types of drinker mostly accounting for alcohol-attributable injuries (4).

The WHO has created a scale AUDIT (5) (Alcohol Use Disturbance Identification Test) to study alcohol profile in users that search for help in big centers. This scale is sensible and identifies the risks and damages of alcohol use, as well as the grade of chemical dependence (5) and has important advantages over other screening instruments since it identifies excessive drinkers who do not meet criteria for alcohol dependence or have not yet experienced actual alcohol-related problems (6).

Since there are very few randomized and prospective studies in literature about the association of facial trauma and the use of alcohol, the purpose of this study was to investigate the impact of alcohol use in facial trauma. The investigators hypothesize that patients with facial trauma have high risk for alcoholism, for this the specific aims of the study were associate AUDIT test with trauma etiology, facial region involved, type of injury and treatment as well as days of hospitalization.

## Material and Methods

To address the research purpose, the investigators designed and implemented a prospective and cross sectional study. The study population was composed of patients presenting facial trauma who came for evaluation at Oral Maxillofacial Surgery Department of Restauração Hospital, between March and December of 2011. The study was approved by the Hospital Ethics Committee.

To be included in the study sample, patients had to have the following inclusion criteria: 1) a definite diagnosis of maxillofacial trauma and detailed description of the physical examination in the period related; 2) definitive treatment already done; 3) a signed informed consent, those who didn´t want to participate were excluded.

Trying to find a relation between disturbs of alcohol use and facial trauma, predictor variables were recorded: trauma etiology, facial region involved, type of injury and treatment, local of fracture and days of hospitalization. The outcome variable collected was the diagnosis of alcohol use disorders. For this AUDIT test (Alcohol Use Disturbance Identification Test) (5-8) was applied to identify risks and damages of alcohol use and chemical dependence. This questionnaire has 10-questions: the first 3 measure alcohol intake (amount and frequency of alcohol consumption); the next 3 refer to alcohol dependence; the last 4 evaluate recent and past issues associated to alcohol consumption (9). Questions one through eight are scored from zero to four. Questions nine and 10 are scored zero, two or four. The maximum score of AUDIT is 40 and a score equal or greater than 8 means high risk for alcoholism (10). This questionnaire was not applied in day of trauma because alcoholic conditions could have interfered in answering it.

Absolute distribution, uni and mutilvaried percentages were made for data evaluation. Pearson´s qui-squared and Fisher´s Exact tests were also used. A model of multiple linear regression was used for determining which types of injury influenced days of hospitalization.

## Results

One hundred patients were evaluated. The patient´s mean age was 33.50 years-old (minimum 17, maximum 89), 48% had between 17 and 29 years old, 28% had 30 to 39, and 24% 40 or more. Most of them were male (86%), 54% were single and 14% never had gone to school.

 Table 1 shows patients distribution according to trauma etiology, affected facial area, type of injury, local of fracture, type of treatment, days of hospitalization and AUDIT classification. As regard to this table, the most frequent etiology was traffic accident (using bicycles, cars or motorcycles) (57%) (out of this 64.91% were involving motorcyles), the extraoral area was most committed (62%), the most frequent type of injury was fractures (78%) and the most affected bone was the mandible (36%) followed by the zygoma (34%). More than half of the patients (53%) had their treatment surgical. 38% had their discharge from hospital right after the first attendance. And the AUDIT most frequent answer was “moderate use” (46%) and use at risk (39%).

**Table 1:**
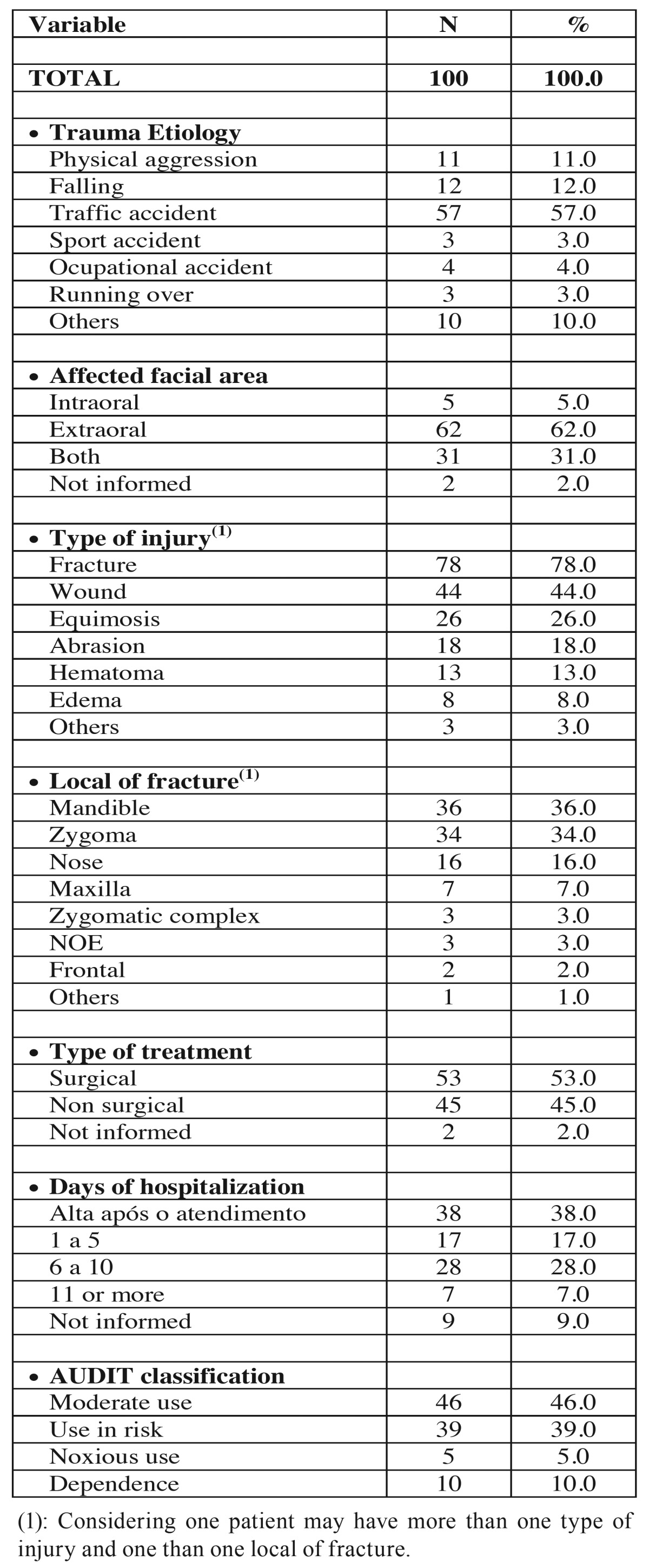
Patients distribution according to trauma etiology, affected facial area, type of injury, local of fracture, type of treatment, days of hospitalization and AUDIT classification.

 Table 2 shows significant differences between the use of alcohol (AUDIT) and the trauma data. The only variable with significant difference was hematoma (*p*=0.003).

**Table 2:**
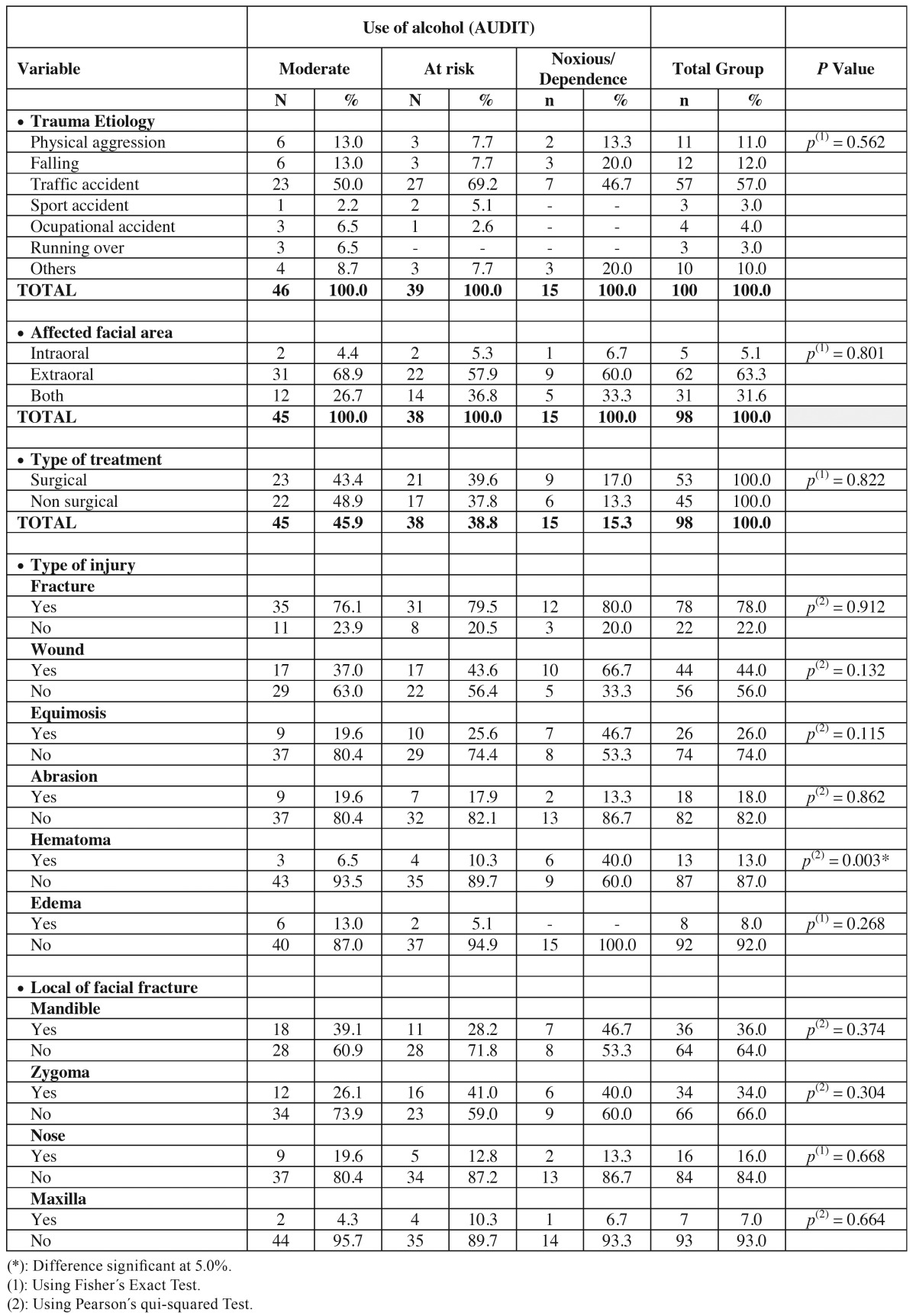
Evaluation of trauma according to the use of alcohol (AUDIT).

 Table 3 shows a significant association between all variables of days of hospitalization and alcohol use (*p*=0.005). It was observed that 35% of the patients with noxious dependence to alcohol stayed more days in the hospital, meanwhile no patient with moderate alcohol use stayed this long. Furthermore when comparing the discharge from hospital after first attendance patients with moderate alcohol used had greater percentage (46.3%) than with noxious use (21.4%).

**Table 3:**
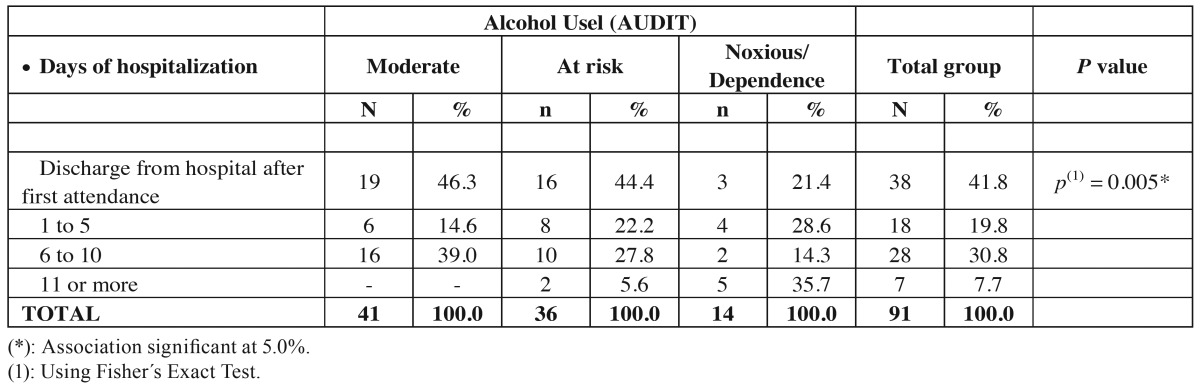
Evaluation of days of hospitalization according to alcohol use (AUDIT).

According to the days of hospitalization it was observed that most of the patients with fractures (38.4%) and equimosis (52%) were in the hospital for 6 to 10 days. Most of the patients who stayed 1 to 5 days had hematoma (53.8%). There was significant differences between type of injury and days of hospitalization for fracture (*p*<0.001), wound (*p*=0.03), equimosis (*p*<0.001) and hematoma (*p*=0.004), meaning that patients with this type of injury stayed longer in the hospital than the ones who did not ( Table 4).

**Table 4:**
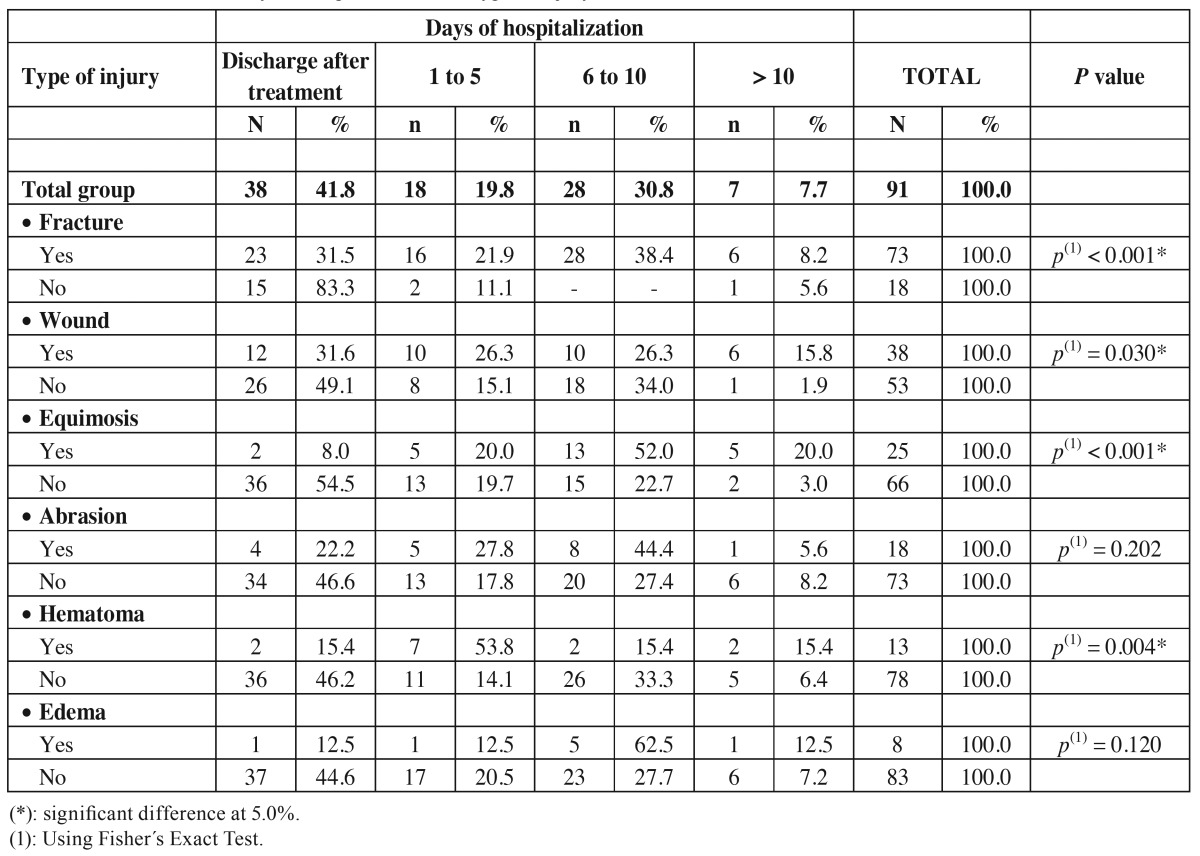
Evaluation of the days of hospitalization and type of injury.

 Table 5 shows the multiple linear regression results for days of hospitalization related to type of injury such as bone fracture, wound, equimosis, abrasion, hematoma and edema. It was observed that all type of injury had significant difference (*p*<0.05), except for abrasion and hematoma. The R2 value was 0.417 and using ANOVA for regression the model is significant (*p*< 0.001) for days of hospitalization. Furthermore the greatest standard coefficients were for equimosis (0.380) and fractures (0.357).

**Table 5:**
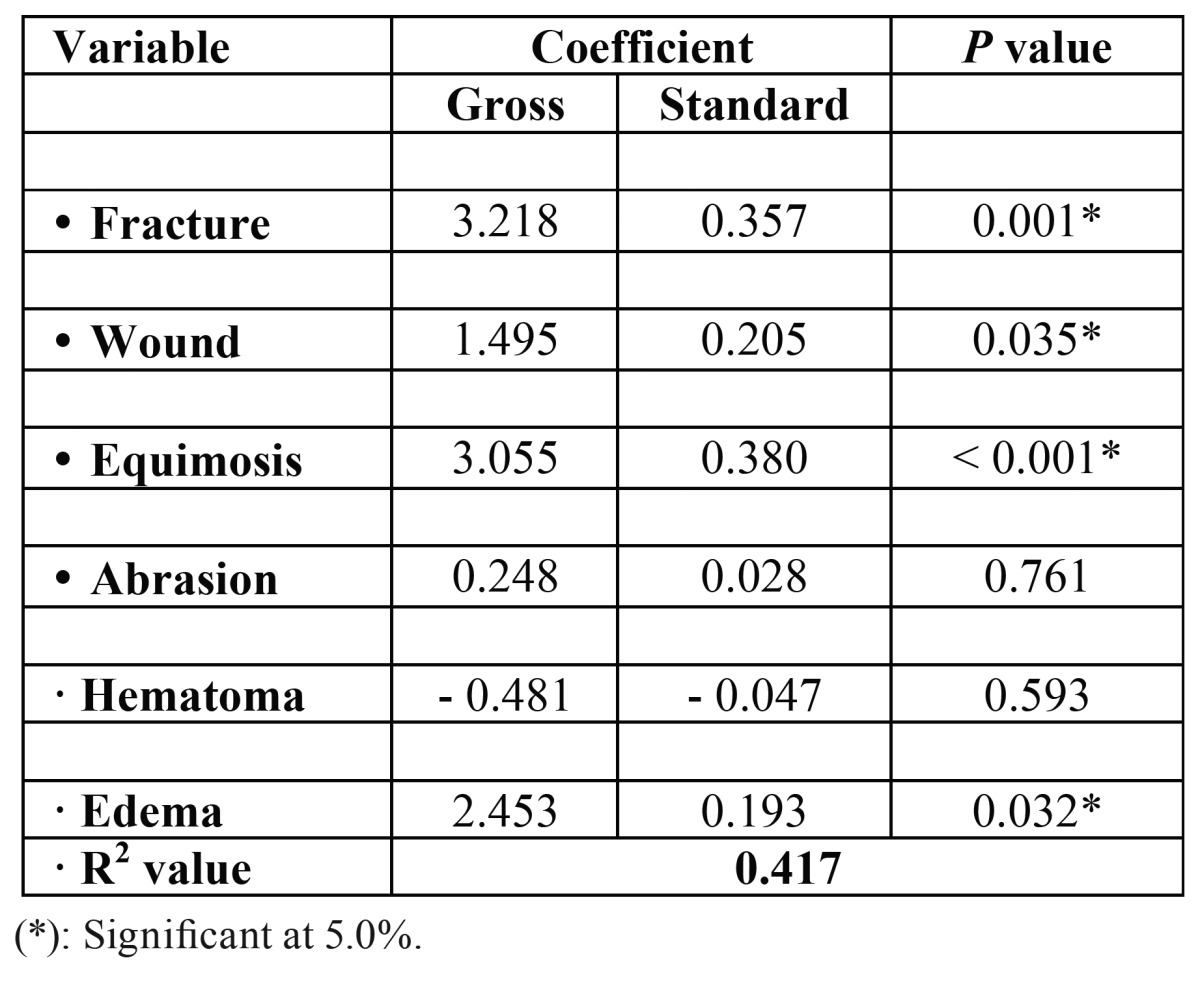
Linear regression results for days of hospitalization and type of injury.

## Discussion

Facial trauma may be considered as one of the most devastating aggression found in health centers and abusive alcohol consumption with association to driving may be a significant factor for causing it. Since there are very few randomized and prospective studies in literature about the association of facial trauma and the use of alcohol, the purpose of this study was to investigate the impact of alcohol use in facial trauma. The investigators hypothesize that patients with facial trauma have high risk for alcoholism, for this the specific aims of the study were associate AUDIT test with trauma etiology, facial region involved, type of injury and treatment as well as days of hospitalization.

Incidence and etiology of maxillofacial fractures vary from country to country (11). In the last 20 years, several studies evaluated the increasing number of facial traumas caused by car or motorcycle accidents associated or not to alcohol (4,12-16). In the eighties, results of a study in Brazil using 450 individuals with facial fractures showed car accidents as main cause (1). Furthermore in a more recent study the causes of facial fractures were car accidents in 25% of cases, motorcycle accidents in 25%, and less frequents were physical aggression in 15% and work accidents in only 1% (17), the mean age was 30.9 years and the proportion of male/female was 3.3:1.21. From 1988 to 1998, a facial fracture retrospective study found the cause as following: 31.8% were caused by car accidents, 22.2% by physical aggression and 18.7% by fire gun shots (18); the main age was 33.50 years and 86% were males. In this study, 57% of the trauma was caused by traffic accidents and out of this total 64.91% were by motorcycles.

Among recent studies associating trauma with body parts injured and use of drugs (19-21), some of the common characteristics found was greater frequency in males and young adults between 20 and 30 years-old (20-22). This is may be due to the fact men are in greater number in the traffic and use more drugs and/or alcohol (22). In agreement to literature this study had 86% of men and were mostly young with age between 17 and 29 (48%). The probable reason why there were very few women in this study is that women with alcohol use disorders have less chance of being violent or arrested or involved in a traffic accident (4,23).

All over the world, around half of fatal traffic accidents are related to alcohol consumption (1,14,20,24). Even though there are very few studies evaluating the relation of alcohol consumption and fatal and non fatal accidents. In this study it was not observed association between alcohol consumption using the AUDIT and trauma etiology (*p*=0.562), but patient victims of traffic accidents were classified as with risk in the scale.

Lee (25), in an 11-years retrospective study of facial traumas, found fractures of zygoma, mandible (angle and condyle) as respectively the most prevalent. de Matos *et al.* (26) found condyle fractures (28%) as the most prevalent, followed by mandibular body (25%), simphysis and parasimphysis (22%). On the other hand, in this study mandible was most affected (36%), followed by zygoma (34%) and nose (16%). Furthermore facial fractures were present in 78% of cases, 44% were wounds, 26% equimosis and 18% abrasion. But no significant relation was found between alcohol consumption using AUDIT and facial fractures, equimosis or abrasion.

This study used a questionnaire to identify the risks and damages of alcohol use, as well as the grade of chemical dependence (5), however there is no way to confirm if the patient were alcoholic right before trauma which could be done by an intoxication test in the moment of patient´s arrival in the hospital. Nonetheless this research studies the relation between facial trauma and alcohol use using a questionnaire, which has not been done often in literature.

Since there is a strong association between alcohol consumption and injury (2) and very little is known about drinkers (4), this study plays an important role on diagnosing the types of drinkers related to unintentional and intentional facial trauma injuries. The investigators used the Alcohol Use Disorders Identification Test (AUDIT) (5) questionnaire that has important advantages over other screening instruments since it identifies excessive drinkers who do not meet criteria for alcohol dependence or have not yet experienced actual alcohol-related problems. Screening for alcohol consumption

among patients in primary care carries many potential benefits. It provides an opportunity to educate patients about low-risk consumption levels and the risks of excessive alcohol use.

Adoption of restrictive laws for consuming alcohol and drugs is known for reducing traffic accidents (27). In Brazil it was created two main federal law to prohibit and mainly punish the combination of alcohol and driving which effectively reduced number of victims (15,16). Adding to this the use of seat belt is considered the most efficient method for reducing gravity of accidents, it is known to reduce in 40-65% the risk of death (26,28). Even then, if this frequency is maintained it is estimated that until 2020 annual proportion of deaths and deficiencies as a result of road accidents will increase up to 60%. As a consequence it will be the third in a list of main diseases and trauma causes of the WHO (6).

Oral maxillofacial surgeons must be involved in the daily preventions of these injuries, giving to patients orientations about the risks and morbidity related to the abusive use of this substance. This orientation is mandatory since maxillofacial trauma associated to alcohol consumption tends to relapse.

Facial deformation caused by this type of trauma may leave its victim more sensitive and vulnerable to the learning moment, which occurs a little while after. In this state of vulnerability is more probable patients recognize and accept their issues with alcohol consumption, being more receptive to therapy and accepting to reevaluate their drinking habits.

Conclusions

In this study it was not observed association between alcohol consumption using AUDIT and trauma etiology, but patient victims of traffic accidents were classified as with risk in the scale. Most of the trauma were caused by traffic accidents using motorcycles and occurred in young aged men.
